# Dr. Prout on Injecting the Blood-Vessels

**Published:** 1813-08

**Authors:** Wm. Prout

**Affiliations:** Leicester Place, Leicester Square


THE
### Medical and Physical Journal.

2 OF VOL. XXX.] AUGUST, 1813. [NO. 174.

*To the Editors of the Medical and Physical Journal.*

GENTLEMEN,

BEING engaged in a subject which at present occupies the greatest portion of my time, I have been induced to publish the following observations, with the hope that some one who may have greater abilities and opportunities than myself may pursue them: should this be deemed a sufficient apology for their imperfections, the insertion of them in your valuable Journal will oblige, Gentlemen,

*Leicester Place*, Your obedient Servant,

*Leicester Square, June* 10, 1813. WM. PROUT, M.D.

THE art of injecting the blood-vessels of animals has received very little improvement since the time of the celebrated Ruysch; indeed it may be said to have been at once invented and matured by that indefatigable anatomist, as few since him have even equalled, much less surpassed, the perfection and beauty of his preparations. As commonly practised, however, it obviously labors under imperfections which in many instances render it useless, there being innumerable parts in the bodies of animals which will not admit the coloring particles of the blood, much less those of common injections; and yet in a physiological point of view these are of the utmost importance, as in them take place many of the most interesting operations of organic life.

I shall not stay to inquire how far the structure of a part influences its operations, nor do I mean to contend that a knowledge of such structure, could it be obtained, would lead to a discovery of the nature of its operations: on the contrary, in the present state of our knowledge, I do not think it would; but facts of every kind are important, especially to the philosophical inquirer; and hence I flatter myself, that to him at least, the following observations, though imperfect, will not prove wholly uninteresting.

It is now some years since the notion of precipitating the coloring matter from a state of solution in the vessels themselves first occurred to me; some difficulties, however, which presented themselves, and more especially want of leisure, prevented me from putting them in practice till within the last three or four months: my first experiments, which were made upon the most difficult organ to inject, viz. the eye (of the ox) were attended with considerable success, the vascularity of most of its transparent parts having been plainly demonstrated.

The coloring particles of injections, it is obvious, should be opake, but to be opake they must be solid;* and solids of every kind, however finely comminuted, will not, as before observed, enter the vessels of transparent parts; and even supposing a fluid could be obtained, holding a sufficient quantity of coloring matter in solution, yet it cannot be fixed by the common means; for I have reason to believe, from some experiments, that a solution of isinglass, of such a strength as just to gelatinize when cold, will not enter them. Injections, therefore, capable of penetrating these infinitely fine vessels, must, in the first place, be fluid as water; and, secondly, like water be inert with respect to animal matter, otherwise they will act on the parts, and, by destroying their organization, defeat their own purpose.

The great difficulty of finding fluids possessed of these properties, and at the same time holding coloring matter enough in solution, was the grand objection which first presented itself when the above-mentioned plan originally occurred to me: almost all the metallic salts, some of which are otherwise well adapted for the purpose, are necessarily excluded from their well-known properties of acting on animal matters: the two which promised most fairly, were the prussiate of potass, and a solution of the red sulphate of iron,

* Mercury perhaps is, strictly speaking, the only *opake* fluid, and this accordingly is often employed for minute injections; the impossibility of fixing it, however, added to its weight, and the strong attraction of its particles for each other, by which they coalesce into globules, and thus block up or burst the minute vessels, very much limit its use. I doubt much also if oils, from their strong antipathy to the watery fluids of the parts, can be made to enter them; and if they could, perhaps there is no method of coloring them so as to render them sufficiently effective. I may here mention a method of common minute injection, which I have employed with considerable success when the parts are capable of bearing the requisite temperature without being injured; this consists in using, instead of size, the serum of blood, or, what is perhaps better, the whites of eggs, a little diluted with water; the coloring matter is to be added to these in the usual proportions and thoroughly mixed, and the part when injected to be thrown into water nearly boiling hot, so as to coagulate the albumen. The easiest way of breaking down the whites of eggs and rendering them smooth, is to force them by means of a syringe through a piece of linen three or four times thick; this repeated once or twice will render them perfectly smooth.

and and accordingly it was with these my first experiments were made. I need scarcely add, that when solutions of these two salts come in contact with each other, a beautiful blue precipitate takes place, which is in fact the well-known pigment called prussian blue. Besides these, I have used for transparent parts solutions of the muriate of barytes, and an alkaline sulphate; also a concentrated filtered solution of the coloring matter of cochineal and the muriate of tin; the former producing a white precipitate, the latter a red one, or, in fact, carmine.

With respect to the strength of these solutions, I have generally used the prussiate of potass, the muriate of barytes, and the infusion of cochineal, in nearly a saturated state, especially the last; the two former, when in a state of saturation, may be diluted with about an equal quantity of distilled water. The other solutions must be much weaker, especially the sulphate of iron, a few drops of a saturated solution of which to the ounce of distilled water will be found sufficient. The best way is, however, to ascertain previously by experiment the strength of the three last injected fluids, which must be such as just to produce a full precipitate, and no more.

I do not mean to say that these are the only solutions that can be made use of for this mode of injection; no doubt others, and perhaps preferable ones, might be pointed out; but I confine myself to these, as they are the only ones I have submitted to experiment. As to the steps preliminary to injection, they are nearly such as commonly observed; I would, however, recommend the pipe to be fixed, when the artery is small, before the part be soaked, as it has been found no easy matter to do it afterwards. It is then to be put into water of the temperature of from 90 to 100, and suffered to remain till thoroughly heated, when the solutions previously heated to the same temperature, and contained in different syringes adapted to the same pipe, are to be injected one after the other. As both the fluids employed have a share in the production of the coloring matter precipitated, it may seem immaterial which of the two precedes the other; I have, however, succeeded best by first injecting the prussiate of potass, the muriate of barytes, and the colored solution of cochineal, and afterwards the solutions before-mentioned adapted for precipitating each respectively. Very little force is to be used, and time allowed for the fluid to penetrate every part; this is especially to be observed in injecting the second or precipitating fluid, which cannot be well done too slowly. If the part is intended to be preserved, a common minute injection made of size colored

 with with a similar pigment, may follow in the last place, which, after forcing out the others from the larger vessels, will occupy their room, and thus complete the preparation. After injecting the first of the two fluids, I have occasionally dissected the part and thrown it into the second, which penetrating precipitates the first; but this method I do not recommend except in particular cases, or where the operation has been unsuccessful, as there is always a confusion and indistinctness, and the parts look rather dyed than injected. The white injection, as before observed, is only applicable to transparent parts, or preparations to be dried and kept in oil of turpentine. With respect to the red injection, it is perhaps the most imperfect of the whole, though it is capable of the most extensive use.

It may not be amiss here to make a few observations on the imperfections of minute injections in general. In the first place, the most frequent terminations of exhalent arteries must obviously be into the vacuities of the cellular membrane; injections therefore which are capable of penetrating these vessels, must necessarily enter these vacuities, and be collected there, and thus cause a sort of general extravasation, which makes the parts appear infinitely more vascular than they are in reality: this is a great imperfection in all minute injections, which it is impossible to obviate, and unluckily it increases as the injection becomes more minute. Another grand defect is the impossibility of injecting the arteries or veins alone, without filling both at the same time. While these imperfections, however, are common to all minute injections, there is one which is peculiar to ours, and which perhaps, with every precaution, cannot be entirely obviated, though it may be considerably lessened: this is, that the coloring matter precipitated by the second solution is apt to block up the vessels, and render them impervious to its further passage, so that in many cases it never enters at all a considerable number of the vessels, and the part has of course the appearance of being uninjected. The only mode of combating this difficulty, is carefully to adjust before-hand the strength of the precipitating fluid, and taking care that it be not too strong, injecting it also very slowly, and using very little force. This defect lessens considerably the probability of success with our plan, though from its greater susceptibility in other respects, the chance may be considered as about equal with the common minute injection; at all events we may, after having injected the first solution, dissect the part, and throw it into the second, and thus ascertain, at least, if it be vascular or no; a fact which, with respect to colorless and transparent parts (to which which only this method is chiefly applicable) can, I presume, be obtained in no other way.

As remotely connected with this subject, I would here recommend caoutchouc, or Indian rubber, as a covering for glasses containing preparations preserved in spirits, &c. It may be softened by means of boiling water, in such a manner as to be easily stretched over the mouth of the glass as thin as parchment. It must be observed, however, that oil of turpentine, which is often used for these purposes, acts on this substance; and hence in this case a piece of bladder, as usual, should be placed in contact with the oil, and over this the caoutchouc. From some imperfect experiments, I have been led to believe, that the evaporation of most volatile fluids is prevented better by this substance than almost any other.

I shall close this imperfect sketch by offering a few remarks upon the ultimate vascularity of the eye.

The vessels of all parts of this organ appear to communicate freely with one another; the part least connected with the rest is the retina, and this is supplied by its own proper artery; a successful injection, therefore, by one of the ciliary arteries, will commonly be found to extend to every part, except the retina, and this, if at all injected, will be only partially so at its interior part. Thus at different times I have succeeded in coloring the cornea, the hyaloid membrane, the capsule of the lens, and once I flattered myself the external coat of the lens itself; on each of which in order I shall now make a few observations.

The *cornea* is known to be composed of lamina, between, and in the substance of which, during life, a fluid is probably exhaled, which preserves that exquisite transparency necessary to the proper performance of its functions: externally it is covered by the tunica conjunctiva, and internally by the membrane of the aqueous humor, both of which appear to differ in their nature, especially the latter, from the cornea itself. These membranes seem to be without proper vessels, and to be analogous to the cuticle, being pierced, particularly that of the aqueous humor, by numerous exhalents. This opinion has been inferred from the fact that the tunica conjunctiva, even after the most successful injection, was very little colored, and that upon its being removed a much greater degree of color appeared immediately below it; the same was also the case with the membrane of the aqueous humor, though it differed from the conjunctiva in seeming to the naked eye to be highly injected: this appearance, however, under the microscope, was found to depend upon an infinite number of points of color, which was considered to be the injection lodged in the mouths of its numerous exhalents,

 at at least the appearance could not probably be explained in any other way. The most vascular parts of the cornea then seem to lie immediately under the membranes which cover its two surfaces, whilst its central part is apparently much less furnished with vessels. Mr. Astley Cooper, to whom I took the liberty of sending a specimen, suggested that the coloring matter might be rather supposed to be lodged in the vacuities of the cellular texture, and this opinion is highly probable. I have, however, been ready to believe, that, with a proper light, (not transmitted, but falling obliquely from above,) I have seen the vessels by the aid of a high magnifier; but I do not vouch this for certain, and it will be understood that wherever the vascularity of a part is mentioned in this paper, it is inferred from the color it has acquired from the injected fluid, not that I have *seen* the vessels themselves. I must leave it to others who have better means than myself to decide if this inference be correct.

The *capsule of the lens* is supposed to be composed of two lamina, one proper and internal, and another external, formed of a reflection of the hyaloid membrane; between these two there probably lies a plexus of vessels, which send off numerous exhalents, that penetrating the internal lamen, secrete the humor of Morgagni: these are inferences I have drawn from having repeatedly seen the capsule injected or colored, and the humor of Morgagni stained and more abundant than usual. With respect to the lens itself, there is certainly a vascular connexion between it and its capsule, if not all over its surface,* at least at its circumference; once I imagined I had injected its external coat, I have many times seen it colored, but could always account for this by supposing that the coloring matter was rather deposited on its surface from the humor of Morgagni. It is however proper to observe, that in no case whatever have I seen it colored internally, though my experiments have been particularly directed to this object.

The *hyaloid membrane* I have many times seen colored in a greater or less degree, and am decidedly of the opinion, that in the adult state at least it derives all its vessels from the great arterial communication, situated a little behind the ciliary ligament, and not from the retina, as usually stated: my reasons for this opinion will be best stated when I speak of the retina; in the mean time I shall merely relate what

---

* I have twice observed after injection the capsule of the lens adhering so firmly to the tunica arachnoidea, that one could not be removed without the other; the humor of Morgagni in these cases was of course wanting, though there was no appearance of disease.

has has occurred at different injections. The anterior part was in general much more highly colored than any other, especially the striæ, situated over the canal of Petit, where this membrane is connected with the ciliary processes. In one instance, after using the muriate of barytes, a portion of this membrane, reflected internally to form its cells, was seen beautifully injected; it had much the appearance of the zig-zag fracture in a piece of crystal, and extended in a direction from the edge of the capsule through the centre of the vitreous humor towards its posterior part. In this case the injection had failed in every other part, so that it was elsewhere quite transparent. The vitreous humor itself I have occasionally seen tinged.

The *retina*, as before observed, is chiefly supplied by its own artery, which however has communication at its anterior part with the general vascular system of the eye. It is known to be composed of two lamina; an internal one, consisting of little more than a plexus of vessels, and an external one, very analogous to the medullary substance of the brain. I have succeeded twice in injecting this part of the eye with the muriate of barytes; there did not appear to be the least communication whatever between it and the vitreous humor, but its vessels seemed to terminate almost entirely in its external or medullary coat, under the form of innumerable penicilli, which, from their proximity to one another, appeared almost to occupy its entire substance. It may here be observed that, probably owing to the injection escaping by exhalents, the prints of the vessels of the retina could be plainly traced on the hyaloid membrane, but they could be easily washed off with a little water, and thus evidently did not enter its substance: it is probable, therefore, that the artery of the retina exhales a fluid between it and the hyaloid membrane; nothing, however, like the central artery* running from the retina through the vitreous humor to the capsule of the lens, of which so much has been said, was ever perceived; I believe, therefore, that in the adult state at least, it has no existence.

All the above observations are to be understood to have been made upon the eye of the ox: those of an individual not too old should be chosen; and I may here mention a curious circumstance, viz. that with those eyes which looked remarkably fair, and in which the cornea was unusually

* Since the above was written I have procured the eyes of a young calf, in one of which I thought I could trace the remains of the central artery; but as nothing of the kind was perceivable in the other, I am inclined to believe it was only an accidental appearance.

transparent, transparent, I have uniformly never succeeded; such eyes have a distinct character, by which they are easily distinguished, and of which the above-mentioned are the prominent traits; but I am totally unable to account for the fact.

Such are the chief remarks I have to offer on that part of the vascularity of the eye named by Bichat the capillary; and, though their *utility* may be called in question, yet they may perhaps afford at least amusement to the curious, and induce him to pursue them. Should this not be the case, I may possibly at my leisure resume the subject, and communicate the result of some experiments already performed, and of others intended to be made, with the view of ascertaining the functions of some parts of this important organ.

